# Multimodal microscope for optical coherence microscopy, tomography, vibrometry, and two-photon microscopy in the living mouse cochlea

**DOI:** 10.1117/1.JBO.30.10.106005

**Published:** 2025-10-16

**Authors:** Clayton B. Walker, Michael J. Serafino, Patricia M. Quiñones, James B. Dewey, John S. Oghalai, Brian E. Applegate

**Affiliations:** a Texas A&M University, Department of Biomedical Engineering, College Station, Texas, United States; b University of Southern California, Keck School of Medicine, Caruso Department of Otolaryngology – Head and Neck Surgery, Los Angeles, California, United States; c University of Southern California, Alfred Mann Department of Biomedical Engineering, Los Angeles, California, United States; d University of Southern California, Ming Hsieh Department of Electrical and Computer Engineering, Los Angeles, California, United States

**Keywords:** optical coherence tomography, optical coherence microscopy, biophotonics, microscopy

## Abstract

**Significance:**

Our understanding of mechanotransduction in mammalian inner ears remains incomplete, in part due to imaging limitations: current systems cannot simultaneously provide high-resolution images needed for subcellular analysis and the deep focus required for structural mechanics. Optical coherence tomography (OCT) enables structural and vibrational imaging through the bone of the intact cochlea in models such as mice, supporting studies of cochlear mechanics in animals with functional hearing. However, capturing both cellular (<10  μm) and structural (>200  μm) details requires rapid switching between optical configurations with numerical apertures ranging from 0.13 to 0.8. A spectral-domain OCT system combined with two-photon fluorescence microscopy (TPM) and interchangeable objectives could overcome this challenge, enabling high-precision vibration and fluorescence imaging across multiple scales in a single experiment.

**Aim:**

We aim to develop an integrated OCT and two-photon microscope optimized for imaging the morphology and function of the cochlea.

**Approach:**

We integrated a custom SD-OCT/TPM system into an upright microscope with a high-precision stage for animal positioning. The system uses two tunable liquid lenses to form a beam expander, enabling dynamic adjustment of the beam diameter at the back aperture of each objective. This optimized light throughput and maintained a high signal-to-noise ratio (SNR) across all objectives. In addition, we automated optical adjustments to facilitate seamless imaging with a wide range of objectives.

**Results:**

For each objective, we measured the SNR difference between a beam expanded to match the largest back aperture and a beam adjusted to match the back aperture of the objective. Except for the 4× objective, the measured SNR improvements closely matched theoretical predictions. Using four selected objectives spanning the required numerical aperture (NA) range, we successfully imaged excised murine cochlea samples, obtaining relevant structural information across scales. In living murine models, we used TPM to locate fluorescent outer hair cells and make vibrometry measurements through the round window membrane. We found that hair cells, the basilar membrane, and the reticular lamina moved in phase in response to a 70 kHz stimulus at 90 dB SPL, consistent with expected cochlear mechanics.

**Conclusions:**

Automation and optimization of the optical system enabled seamless multiscale imaging of the murine cochlea, providing high-quality morphological, functional, and two-photon fluorescence images. The dynamic adjustment of the beam diameter within the microscope was essential for maintaining high SNR across a wide range of numerical apertures.

## 1 Introduction

Optical coherence tomography (OCT) has quickly become an essential tool to assess both the structure and function of the cochlea because of its ability to noninvasively image through the otic capsule and along the depth of the sensory epithelium. Morphological studies using OCT have assessed the cellular architecture of the organ of Corti (OoC),^1^^–^^3^ along with the changes that occur after noise or blast trauma,^4^^,^^5^ such as endolymphatic hydrops (ELH). Functional studies have combined high-resolution cross-sectional images of the cochlea with vibrometry measurements to study traveling wave mechanics^6^^,^^7^ and amplification.^8^^–^^11^ Although these studies have given us insight into the sound transduction process that occurs in the cochlea, most of the physiological studies lack the resolution to measure subcellular structures such as hair bundles, the hair cell organelle responsible for mechano-electrical transduction, or ribbon synapses, which excite the auditory nerve fiber. These structures are essential for understanding how the organ-level traveling wave is transformed into sub-cellular motions that initiate the mechanical-to-electrical signal conversion within hair cells. They are also critical for evaluating cochlear changes, such as synaptic terminal (ST), dendrite, or nerve fiber swelling, which occur after trauma. More importantly, to fully understand cochlear signal processing that begins with sound and ends in nerve fiber spiking, it is necessary to monitor nerve fiber activity. Consequently, it would be advantageous to develop an OCT system capable of higher lateral resolution that can simultaneously assess vibrometry and neuronal activity.

Increasing OCT lateral resolution can easily be achieved using an objective with a higher numerical aperture (NA). The benefits of a high lateral resolution system are increased precision in determining the specific structures from which vibrometry is being recorded, including the ability to measure motion and morphology of subcellular structures. However, the cost of a high NA system is the loss of axial depth of field, which could make it difficult to resolve the entire OoC, limiting the ability to orient within the tissue or visualize the entire structure of an object of interest. Current OCT systems used for research in otology are typically limited to providing either high NA microscopic images on the cellular level (OCM) or low NA images of the cochlea at a macroscopic (organ) scale. This means that different imaging systems are needed to get a complete understanding of the complex cochlear mechanics that involve both microscopic and macroscopic processes, requiring multiple experiments across several animals. And still, the output response of the cochlea, the auditory nerve fiber spiking, is not measured. Although OCT has the capability to measure nerve fiber activity,^12^ the recent availability of genetically encoded fluorophores^13^ such as green fluorescent protein (GFP) and tandem dimer Tomato (tdTomato), along with indicators, such as genetically encoded voltage indicators (GEVIs)^14^ and genetically encoded calcium indicators (GECIs)^15^ that allow for targeted delivery, morphological reconstruction, and recording of the spatiotemporal patterns of neural activity, suggests that two-photon fluorescence microscopy (TPM) could be used in parallel with OCT/OCM to visualize fluorescent proteins targeted to subcellular structures and hair cell, or nerve fiber activity.

We designed and constructed a custom microscope to achieve lower-resolution organ-level imaging with OCT, higher-resolution subcellular imaging with OCM, and *in vivo* TPM for *in vivo* imaging of fluorescent proteins. The system incorporates a tunable beam expander to match the back aperture of the range of objectives used, helping to optimize the signal-to-noise for OCT/OCM. This multimodal system was used to image the cochlea from a transgenic mouse model (${\mathrm{Atoh}1}^{\mathrm{CreERT}2\text{-}\mathrm{tdTomato}}$; ${\mathrm{Tau}}^{\text{EGFP}}$) that has a different reporter fluorophore in hair cells (TdTomato) and nerve fibers (EGFP). Our custom tunable OCT/OCM and TPM microscope was also used to assess vibrometry in a transgenic mouse model (Myo15CreXAi6), where hair cells are labeled with a bright green fluorescent protein (ZsGreen1). This imaging system has the flexibility needed to make morphological and physiological measurements of cochlear structure and function at the macroscopic and microscopic level, with the potential to visualize subcellular organelles, specific cell populations, and possibly measure their activity.

## 2 Materials and Methods

### 2.1 Microscope Features

The custom OCM/OCT microscope is built around a Nikon Eclipse LV100 body with integrated TPM and a tunable beam expander that was used to control the beam diameter at the back aperture of each of the four objective lenses we would need for future experiments. The beam diameter needed to be controlled because we wanted to fill the back aperture of each objective with minimal overfill to maintain a high signal-to-noise ratio (SNR) across all the objective lenses. The objective back apertures varied between 9 and 22 mm, so changing the beam diameter to match the back aperture of the respective objective lens was the only way to maximize the OCT/OCM SNR and lateral resolution of each objective.

**Fig. 1:**
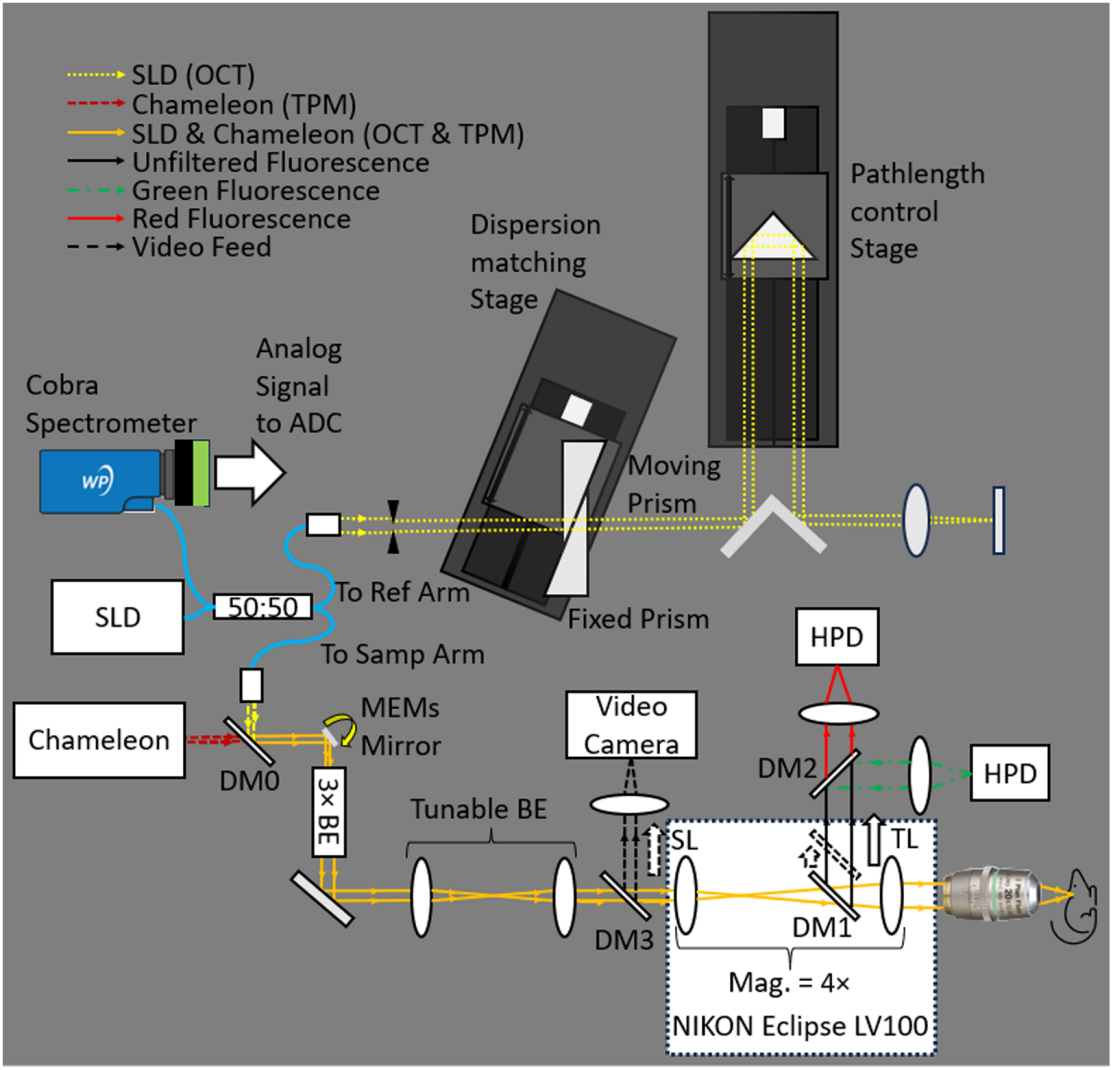
General diagram of OCM-TPM optics. The SLD was coupled directly into a fiber-based 50:50 coupler—where 50% of the light went to the reference arm with the adjustable dispersion matching prisms and pathlength adjustment stage, and 50% went to the sample arm. Starting with the sample arm, DM0 is the long-pass dichroic mirror that combines the TPM laser with the SLD. The 3× BE is the static 3× beam expander just before the tunable beam expander, and DM3 is the long-pass dichroic (735 nm cutoff) to send white light to the video camera. The SL and tube lens (TL) provide the 4× magnification to bring the final beam diameter to the proper size for the objective in use. DM1 is the long-pass dichroic mirror (735 nm cutoff) in the manual optical switch that sends fluorescence light to the hybrid photodetectors when moved into the beam path. DM2 is a long-pass dichroic mirror (585 nm cutoff) to separate the red and green fluorescence channels. The hybrid photodetectors (HPDs) are new photomultiplier tubes (PMTs) that incorporate avalanche photodiodes at the distal end of the PMT vacuum tube as the last stage of a two-stage signal gain system to provide higher gain than standard PMTs with lower noise. The reference arm used a pair of prisms—one attached to a stage and one fixed—to adjust the amount of glass in the reference arm. To adjust the amount of chromatic dispersion in the reference arm that best matched that in the sample arm, the position of the prism on the stage was adjusted to provide a specified amount of glass. The objective lenses were also of different lengths, so we included a second stage to adjust the reference arm optical pathlength length (OPL) to match the length of the sample arm and maintain fringes. The legend in the top left corner indicates the composition of each beam path. Within this system, the reference arm is used solely for the OCT/OCM system, the fluorescence detection optics are used solely for the TPM system, and the rest of the optics are used for both systems.

The Michelson-type interferometer used for OCM and OCT included a broadband superluminescent diode (SLD, Broadlighter, Superlum) with a spectral bandwidth of 173 nm that was centered at 843 nm, providing an axial resolution of 1.95  μm (in air). The TPM source was an 80 MHz femtosecond-pulsed laser (Chameleon Ultra II, Coherent) with an emission wavelength of 930 nm. Both beams were combined at a long-pass dichroic (DM0 in Fig. 1) with a cutoff at 925 nm before reaching the micro-electromechanical system (MEMS) scanning mirror (Mirrorcle Technologies, Inc.), which had a recommended low-pass cutoff scan frequency of 900 Hz. A static 3× beam expander was used to increase the beam size from 1.1 mm to a more manageable 3.3 mm before reaching the tunable beam expander. The tunable beam expander was comprised of two current-driven liquid lenses (EL-16-40-TC-NIR-20D-C, Optotune) separated by 60 cm of free space to allow a maximum magnification of ±5×. The scan lens (SL) and tube lens (TL) provided an additional 4× magnification.

TPM excitation was accomplished with a femto-second laser (Chameleon Ultra II, Coherent) tuned to 950 nm with a pulse duration of 181 fs at the sample. A manual optical switch (Nikon) using a long-pass dichroic mirror with a cutoff at 735 nm (FF735-Di02, Semrock) was placed in the sample arm between the scan lens and tube lens. For fluorescence detection, the dichroic (DM1 in Fig. 1) was pushed into the beam path to reflect the fluorescence toward two hybrid detectors (R11322U-40, Hamamatsu Photonics). The detectors were separated into red and green channels by a long-pass dichroic mirror (DM2) with a 585 nm cutoff. Additional bandpass filters were used to avoid bleed-through between the channels. In case the mouse surgery needed to be revised while under the microscope, DM1 could be removed from the beam path. This would allow white light to travel back up the sample arm to DM3, where a small CCD camera would collect the video image for display on a nearby monitor.

In total, this microscope was built with four different objective lenses in mind for different parts of the *in vivo* mouse experiment as well as planning for future experiments. For OCT imaging of the OoC and Reissner’s membrane, a Nikon 4×, 0.13 NA objective was used with a depth of field (DOF) of ∼50  μm and a field of view (FOV) of 521  μm. For TPM and OCM, three different objectives were investigated as potential solutions. The Nikon 20×, 0.45 NA air objective was preferred with a FOV of ∼110  μm and longer working distance of 8 mm; however, the strong reflection from the intact round-window membrane at the air-tissue interface could produce artifacts and lower SNR, thus reducing the overall image quality. For this reason, a water-dipping Nikon 40×, 0.8 NA objective with a FOV of 60  μm, and a water-dipping 16×, 0.8 NA objective with a FOV of ∼134  μm were both tested as alternatives. The two water-dipping objectives were also advantageous because of their significantly higher NA, which would make them desirable for TPM imaging of sub-cellular structures and dynamic processes.

In principle, if we fill the back aperture of the objective, we should achieve the NA specified by the manufacturer and resolution implied by that NA. However, it is difficult to directly measure either the NA or the point spread function of high NA objectives. However, we can readily measure the axial part of the point spread function by translating a mirror through the focal plane while measuring the OCM signal intensity. We commonly equate the full-width-half-maximum of this measurement with the depth of field (DOF). Table 1 lists all the objectives, the specified NA, measured FOV, measured DOF, effective NA, and lateral resolution. The effective NA was calculated by following Eq. (7) in Ref. 16

$${\mathrm{NA}}_{\mathrm{eff}}=\sqrt{\frac{2\times n\times \lambda }{z_{0}}},$$ (1)

where n is the refractive index of the immersion medium, λ is the wavelength of the system, and $z_{0}$ is approximately the DOF. With the effective NA, the lateral resolution could then be calculated, again following Eq. (7) in Ref. 16 by

$$r_{0}=\frac{0.61\times \lambda }{{\mathrm{NA}}_{\mathrm{eff}}},$$ (2)

where $r_{0}$ is approximately the FWHM lateral resolution and ${\mathrm{NA}}_{\text{eff}}$ is the effective NA calculated by Eq. (1). The calculated ${\mathrm{NA}}_{\text{eff}}$, tabulated on the right of Table 1, is in reasonably good agreement with the manufacture’s specified NA.

**Table 1:** Objectives and performance.

Objective mag	NA	FOV (μm)	DOF (μm)	${\mathrm{NA}}_{\text{eff}}$	Lateral resolution (μm)
4×	0.13	521	168	0.10	5.14
20×	0.45	110	8.8	0.44	1.17
16×W	0.8	134	4.3	0.72	0.71
40×W	0.8	60	3.4	0.80	0.63

**Fig. 2:**
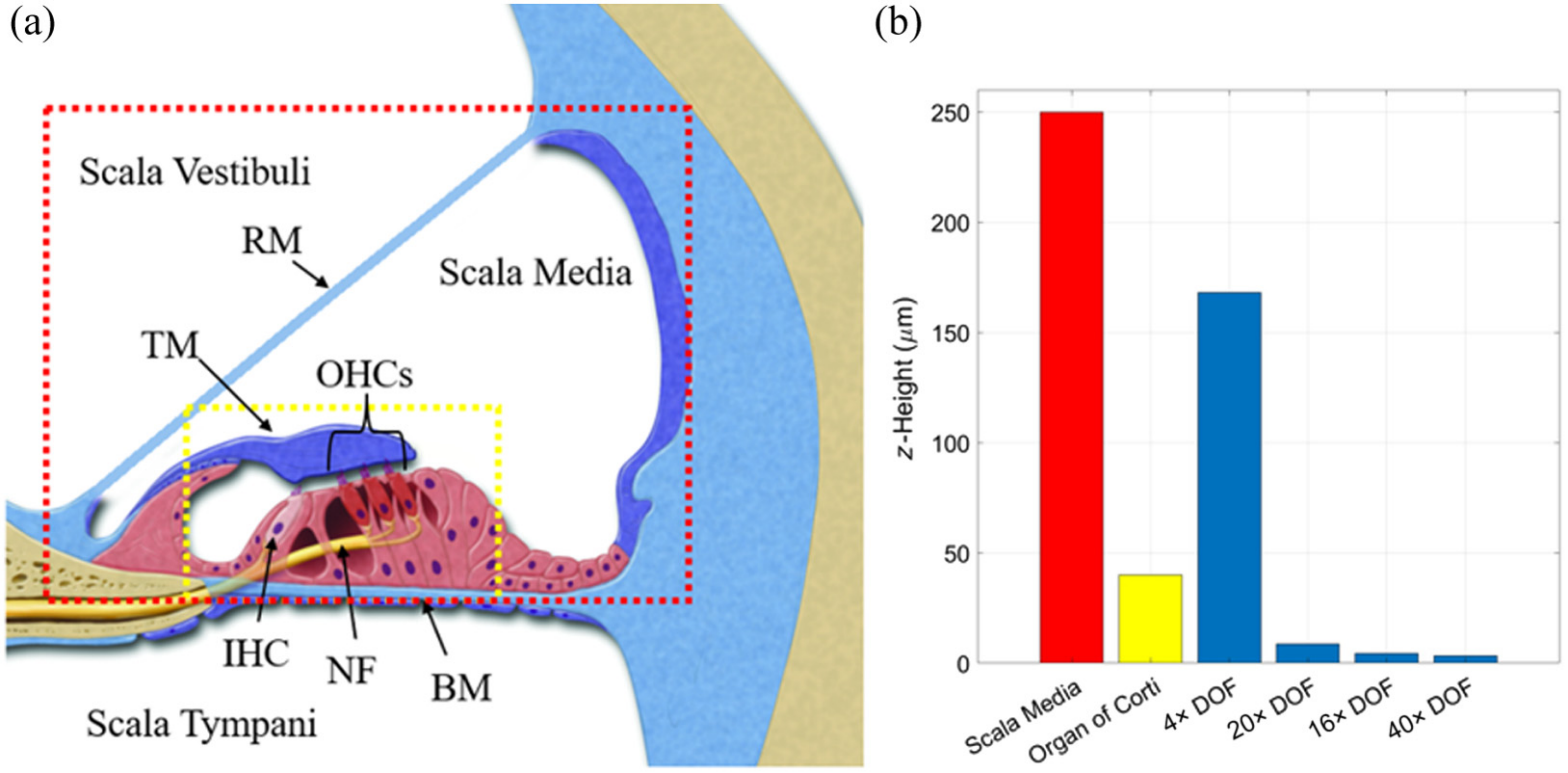
(a) Diagram of the murine cochlea where the red box encloses the entire scala media with Reissner’s membrane indicated by RM and the basilar membrane indicated by BM. The yellow box encloses the entire OoC where TM is the tectorial membrane, OHCs indicate the outer hair cells, IHC indicates the inner hair cell, and NFs are the efferent nerve fibers. As the diagram is not to scale, panel (b) shows the true respective heights of the scala media in red and the OoC in yellow. The DOF of each of the four chosen objective lenses was also included to indicate their strengths when imaging the cochlea.

To further illustrate the need for these objective lenses, Fig. 2(a) shows a diagram of the mouse cochlea with a red box enclosing the entire scala media, which extends from Reissner’s membrane to the basilar membrane, whereas the yellow box encloses the entire OoC. However, the diagram is not drawn to scale. The OoC and tectorial membrane (TM) are shown much larger so that the cellular structure can be seen. As this diagram is not to scale, we also included a bar graph in Fig. 2(b), showing the correct vertical height (z-Height) of the two boxes and the DOF of the four objectives. The 4× objective stands out for its much longer working distance, which would prove useful for imaging the entire scala media to monitor distention of Reissner’s membrane as an indicator of swelling within the scala media (ELH). The 16× and 40× objectives have similar, very short DOF with high lateral resolution for imaging cellular interactions and monitoring nerve fiber swelling within the OoC with TPM. We chose to include the 40× objective because the increased magnification would help with quantitative measurements of swelling of small structures such as the STs near the hair cells. The 20× objective provided a good compromise between high-resolution imaging—making it suitable for TPM—and a sufficiently long DOF to image most of the OoC using OCM for high-resolution vibrometry measurements.

The variety of objective lenses required that the dispersion in the reference arm of this system be adjustable to provide the best physical dispersion correction before applying a numerical approach for fine-tuning. One linear electro-mechanical stage (Zaber) was used to translate a reflective prism, thus increasing or decreasing the reference arm path length, and a second linear electro-mechanical stage (Zaber) was used to slide a prism into the beam path to provide the appropriate amount of glass needed to match the sample arm for each objective lens. A mating prism was kept stationary to prevent dispersion of the beam, and the hypotenuse of each prism was coated with refractive index-matching gel to allow smooth sliding motion between the prisms and prevent damage at their sliding interface. The optimal amount of glass needed in the reference arm was determined experimentally for each objective lens by imaging a mirror in the sample arm and minimizing the width of its corresponding peak in the A-line. This was achieved by tuning the position of the dispersion compensation prism while maintaining the same peak frequency through a corresponding adjustment of the reference arm path length. Figure 1 shows the beam path through the optics in the system, the stages and their movement in the reference arm, and the optical switch with beam paths associated with DM1 being in the beam path or DM1 being absent from the beam path.

The variety of objective lenses also required a variety of back aperture settings to ensure that the maximum effective NA was achieved. To do this, the current was adjusted in each of two tunable liquid lenses (EL-16-40-TC-NIR, Optotune) to provide the required optical power in each lens to achieve the desired magnification. The lenses could provide optical power from −10 to 10 diopters, so the lenses were placed 60 cm apart to provide a tunable magnification that ranged from −5× to 5×. Each setting was determined to produce a beam diameter at the back aperture of the respective objective that would overfill by 1 mm.

Custom software was written to control all processes except the manual optical switch (position of DM1), so switching objectives only required the user to manually switch to the next intended objective on the microscope turret and click the button corresponding to the next intended objective lens. This made switching objectives during an experiment quick and easy because all adjustments were finished automatically in a matter of seconds. The high-precision motorized sample stage (ProScan II, Prior) was also controlled by our software to provide z-stack imaging capability by moving the sample vertically in small increments, allowing the MEMS mirror to perform x to y scans at each position in depth. Users defined the upper and lower bounds of any z-stack while also controlling the number of images or the distance between the images in depth. This allowed our OCM-TPM system to provide 3D images at high resolution, similar to methods used in confocal fluorescence microscopy.^17^

### 2.2 Validation Experiments

To illustrate the need for a tunable beam diameter at the objective back aperture, sets of 10 A-lines from a mirror were collected with each objective lens using the beam diameter that matched the back aperture of the respective objective lens. Then, for comparison, we set the back aperture beam diameter to 21.2 mm to fit the largest back aperture of the set of objective lenses and collected mirror data again with each objective lens. We then calculated the SNR in each case for all objectives using the peak signal divided by the standard deviation of the noise that we collected by blocking the sample arm and acquiring background data.

To validate the performance of the microscope for murine cochlear imaging, we imaged an excised cochlea from a mature ${\mathrm{Atoh}1}^{\mathrm{CreERT}2\text{-}\mathrm{tdTomato}}$; ${\mathrm{Tau}}^{\text{EGFP}}$ mouse, which was created by crossing the Atoh1CreERT2 mouse (JAX#: 007684) with an Ai14 Cre reporter strain (JAX#:007914), and the ${\mathrm{Tau}}^{\text{EGFP}}$ mouse (JAX#:029219). The final result was the ${\mathrm{Atoh}1}^{\mathrm{CreERT}2\text{-}\mathrm{tdTomato}}$; ${\mathrm{Tau}}^{\text{EGFP}}$ mouse (referred to as: AtohTau mouse) that contains two fluorophores for separate identification of hair cells (tdTomato) and nerve fibers (EGFP). The mice were injected with Tamoxifen (Sigma) dissolved in corn oil (Sigma) at a concentration of 100 mg/kg on consecutive days P0 and P1 for expression of the hair cell fluorophore. All experiments were performed according to protocols approved by the Institutional Animal Care and Use Committee at the University of Southern California.

Each objective lens was used to create z-stack images of the excised cochlea with OCM/OCT and TPM in quick succession to simulate the conditions of an *in vivo* experiment and minimize any tissue degradation. The goal of this experiment was to prove that low-NA objectives, such as the 4× and 20×, could be used to locate the OoC, whereas the two higher-NA objective lenses could resolve cellular and subcellular structures such as nerve fibers and synaptic boutons. Figure 4 shows the overlapped OCM and TPM images at the best z-stack depth for each objective, where the fluorescence of the nerve fibers was the sharpest and brightest.

### 2.3 *In Vivo* Mouse Experiment

As a final test of the microscope, an *in vivo* vibrometry experiment was performed through the round window membrane of a Myo15-Cre mouse that was crossed with an Ai6 reporter mouse (JAX#: 007906). This mouse had fluorescently labeled hair cells (ZsGreen1). The mouse was first anesthetized (80 mg/kg ketamine, 10 mg/kg xylazine) and placed on a heating pad to maintain body temperature at 38°C, with supplemental doses of anesthesia given to maintain areflexia. After exposing the skull and fixing it to a head holder with dental cement, a ventrolateral surgical approach was used to access the left middle ear space. The bone under the tympanic annulus was carefully thinned and removed so that the round window membrane could be clearly visualized. The external ear was also removed so that a sound delivery tube could be sealed over the ear canal. The tube was connected to a Fostex speaker (FT17H; Fostex, Tokyo, Japan) with sufficient high-frequency response. Prior to the experiment, sound stimuli were calibrated using a 1/8″ microphone (Type 4138-A, Brüel & Kjær, Virum, Denmark) inserted into the end of the tube.

After visualizing the round window membrane, the 4× objective was used to locate the position of the OoC. TPM was then performed with the 20× objective to target the outer hair cells for vibrometry measurements. The x-coordinates containing hair cell fluorescence (pixels 135 to 152) along the central y-position (150) of the enface TPM image were used as the enface coordinates for OCM vibrometry measurements, which were selected in the collected B-scan image. The final x-coordinate used for the vibrometry data taken from the B-scan image was 144. For vibrometry collection, a 70 kHz pure tone at 90 dB sound pressure level was presented to the ear canal. The entire A-line of the selected x-coordinate was processed to give vibration information at every pixel in depth.

Once we finished taking vibrometry measurements, we quickly switched from the 20× objective to the 4× objective and took several consecutive cross-sectional images of Reissner’s membrane without moving the mouse. This was meant to simulate the experimental protocol used for studying ELH and its relationship to trauma in the OoC.

## 3 Results and Discussion

### 3.1 Validation Experiments

**Fig. 3:**
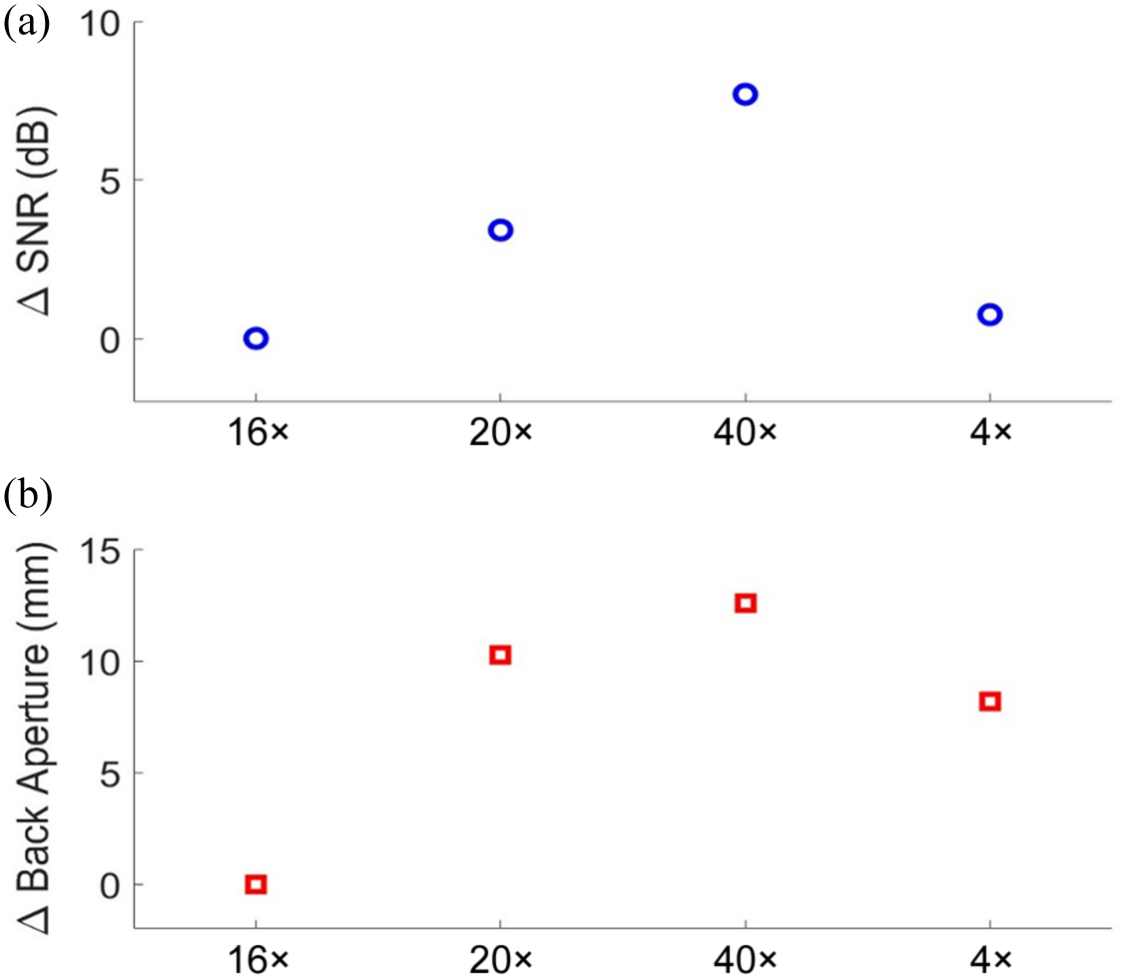
(a) SNR comparison for each objective. Here, ΔSNR represents the difference in signal-to-noise ratio (SNR) between two configurations: one where the beam size matches the back aperture of each objective, and another where a larger beam size—sufficient to fill the largest back aperture (21.2 mm, corresponding to the 16× objective)—is used for all objectives. Blue circles indicate the ΔSNR values for each objective lens under these two conditions. The red squares in panel (b) represent the difference in the beam sizes used to take the measurements for the SNR plot in panel (a).

The differences in SNR from the validation experiment, shown in Fig. 3, were calculated using the following equation:

$${\;\mathrm{SNR}}_{m}-{\mathrm{SNR}}_{u}=\;\Delta \mathrm{SNR},$$ (3)

where ${\mathrm{SNR}}_{m}$ was the SNR of A-lines collected when the beam diameter at the back aperture matched the diameter of each objective’s back aperture, and ${\mathrm{SNR}}_{u}$ was the SNR of A-lines collected with a fixed beam diameter of 21.2 mm, corresponding to the largest objective lens back aperture. The differences between each objective’s back aperture diameter and the fixed beam diameter were also calculated to provide context on beam clipping in objectives with smaller back apertures.

As expected, the SNR increased when the beam diameter matched the back aperture of each objective when compared with the SNR achieved from each objective with the beam diameter set to the largest size. The 40× objective, which had the smallest back aperture, had the largest change in SNR with an 8 dB gain when using the correct beam diameter versus the largest beam diameter. We also saw differences in SNR of 0.7 and 3.5 dB for the 4× and 20× objectives, respectively, which mirrored the change in aperture of the lenses. Tuning the beam diameter to match the aperture of each of the objective lenses can provide significant SNR improvement, given the range of back aperture sizes (8.6 to 21.2 mm) for the set of lenses required for imaging the murine cochlea.

**Fig. 4:**
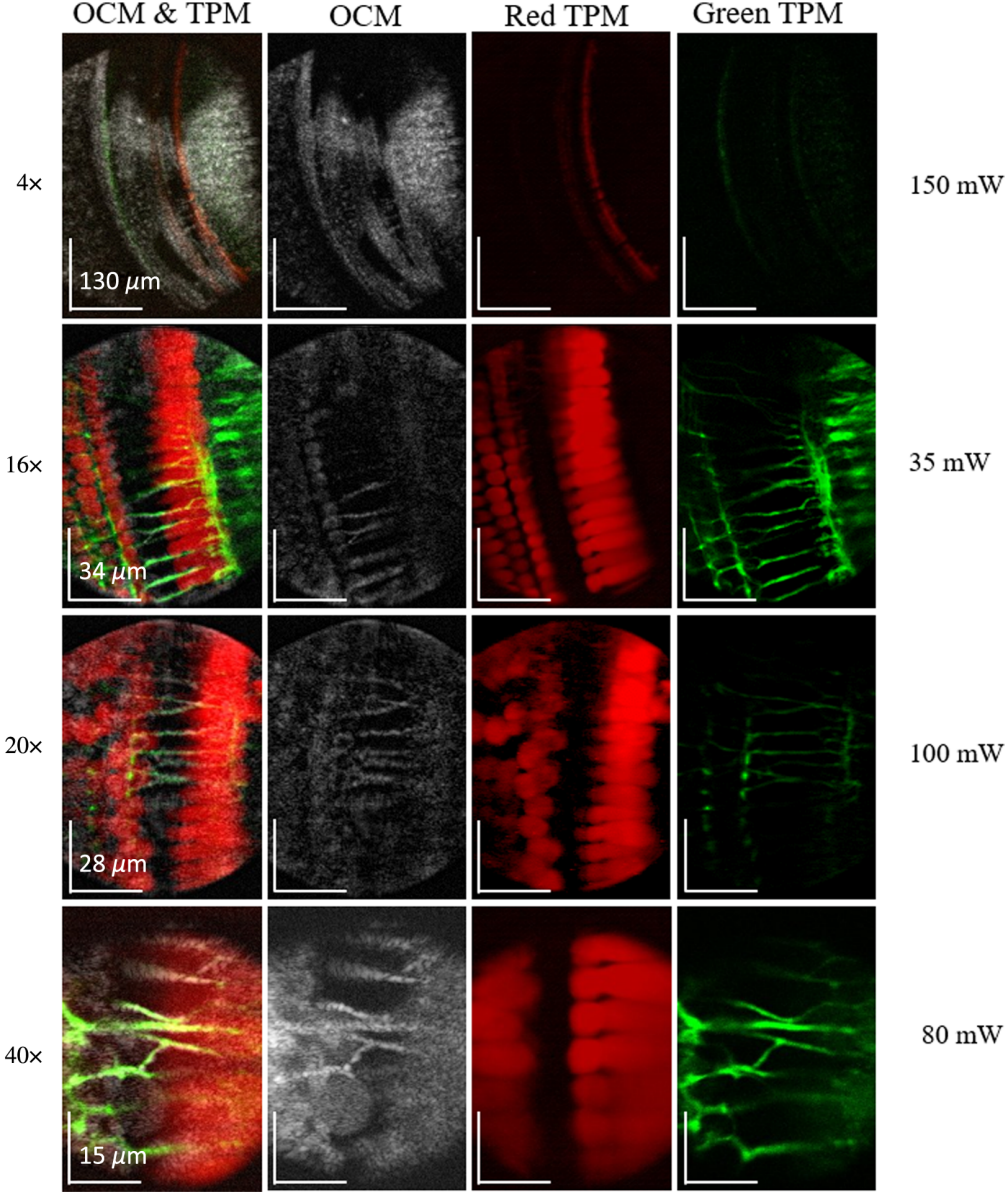
Representative images of an excised cochlea taken from a z-stack where the tissues of interest were in the focal plane of each objective. Each row corresponds to a different objective, as labeled, and each column corresponds to the image type labeled at the top of the column. The far-left column demonstrates the colocalization of both fluorescence channels with the corresponding OCM image. Representative olivocochlear (efferent) nerve fibers are indicated with arrows, inner hair cells are indicated with IHC, and outer hair cells are indicated with OHC. Labels on the right-hand side of the figure indicate the average power of TPM excitation used to get the fluorescent images.

To get a better understanding of the image quality and resolution of the system within the intended sample, we turned to *ex vivo* imaging of a fluorescent-labeled murine cochlea. Localizing the area or position inside the cochlea while visualizing structures such as nerve fibers and hair cells using TPM is critical to properly understand how structure and function change after an insult such as blast or noise trauma. Figure 4 shows the OCM-TPM images collected from the excised cochlea using each objective lens as described in Sec. 2.2. The far-left column of Fig. 4 shows the two fluorescence channels and OCM overlaid to confirm that OCM can be used to find the precise location of a region of interest in the tissue. This means that the user can find the region of interest without using TPM, thus reducing the risk of photobleaching the sample. This is crucial for studying the effects of blast or noise trauma as the resulting ST swelling is a process that takes time to progress, and photobleaching could result when searching for an appropriate imaging location within the OoC. The OCM column further confirms that we can see important landmark structures without the aid of TPM. The nerve fibers can be clearly seen in all but the 4× objective lens—meaning all high-NA objectives perform well in this regard. The final two columns on the right show the red TPM channel, where only the hair cells are visible because they express tdTomato, and the green TPM channel, where the nerve fibers are visible because they express EGFP. The STs are located near the hair cells, so the red fluorescence allows us to fine-tune the imaging region to localize dendrites. All three high-NA objectives were able to clearly visualize the important fluorescent tissues to complete the high-NA task of localizing and monitoring dendrite swelling. The main difference between each of these objectives was the image FOV and lateral resolution. For experiments where both the outer and inner hair cells need to be monitored, the objective providing the larger FOV that shows both in a single image can be used, whereas experiments that are imaging single boutons can use those with a reduced field of view, but better lateral resolution. Because of the ease of moving back and forth between objectives, a combination of objectives that fit the experimental requirements can be utilized.

**Fig. 5:**
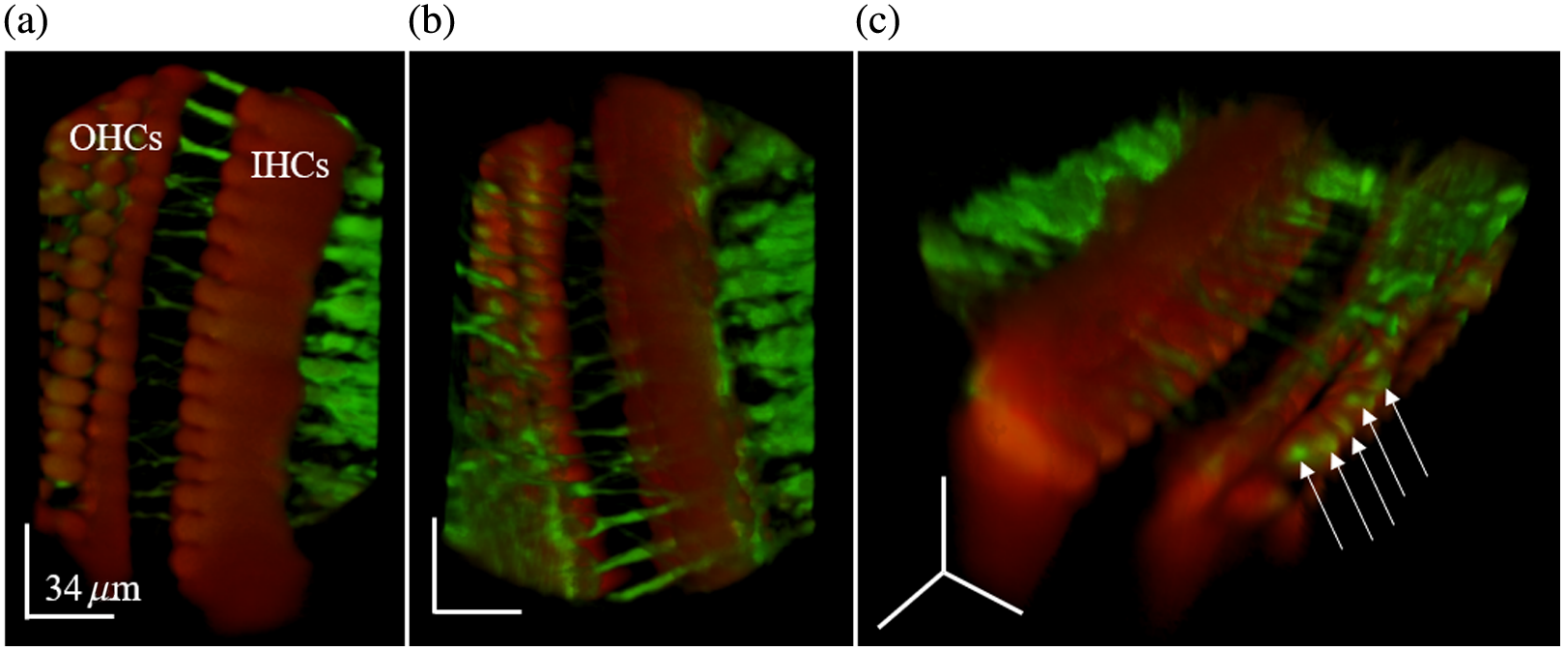
Volumetric rendering of the TPM stack acquired using the 16× objective. (a) View looking down on the top of the hair cells, (b) view looking down on the nerve fiber synapses with the OHCs, and (c) 3D view of 3D volumetric image of both fluorescence channels combined to show the location and morphology of the hair cells (red), nerve fibers (green), and ST synapses with the OHCs (arrows).

To demonstrate the system potential in creating volumetric images of the fluorescent tissues within the mouse cochlea, we combined all the fluorescent images taken in the z-stack from the 16× objective into a volume rendering (Fig. 5). Because the nerve fibers synapse with the basolateral pole of the hair cells where STs normally contact hair cells, the hair cells are shown to be above the level of the nerve fibers in Fig. 5(a), which has been rotated such that the hair bundles of the hair cells face up. When rotated such that the hair bundles point down, as in Fig. 5(b), we can see the nerve fibers that are known to cross from the IHC region through the tunnel of Corti (ToC) and synapse with the outer hair cells (OHCs). These images confirm our expectation of seeing nerve fibers coming across the OoC to synapse with the outer hair cells. Figure 5(c) is a 3D view of the tissue where we can clearly identify STs contacting the basal pole (on top in the image) of the outer hair cells.

To better understand the TPF resolution, we compared OCM and TPF images where the nerve fibers cross the tunnel of Corti (labeled with white arrows in Fig. 4). In this region, the nerve fibers have high contrast in the OCM images. Nominally, the resolution enumerated in Table 1 only applies to the OCM system. However, both TPF and OCM use the same imaging optics; hence, we can expect them to track one another. Cross-sectional analysis of nerve fibers shows that TPF widths are ∼61% to 90% of the OCM width for the three objectives we would normally use for TPF, i.e., the 20×, 16× W, and 40× W lenses. Consequently, multiplying the lateral resolution values in Table 1 by the average, 0.75, should give a reasonable estimate of the TPF resolution.

### 3.2 *In Vivo* Mouse Experiment

**Fig. 6:**
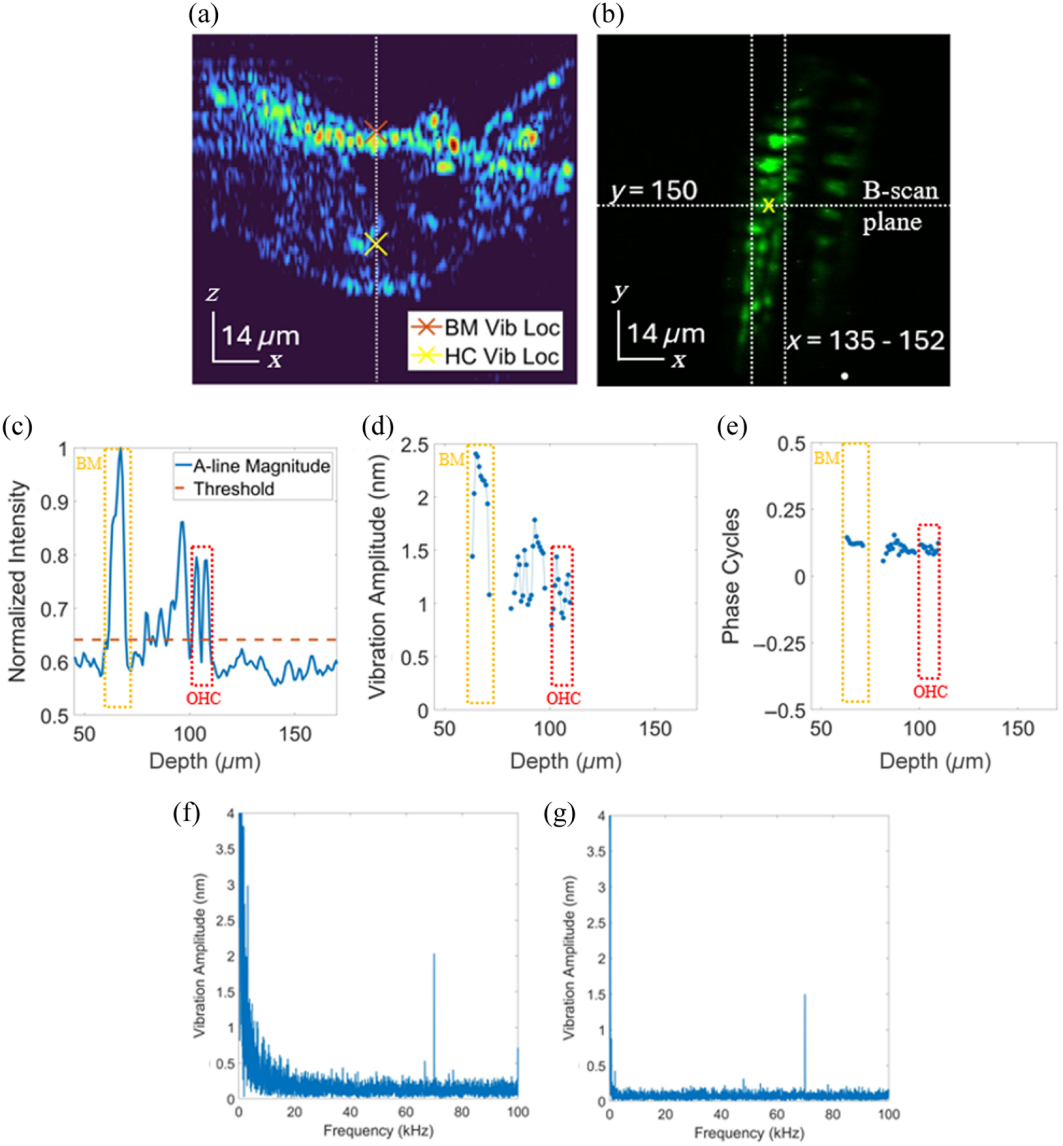
*In vivo* vibrometry of the OoC. (a) Average of 50 B-scan images of the OoC through the RWM using the 20× objective and showing the two locations (BM, OHC) in the A-line from which vibrometry was recorded. Panel (b) is the enface fluorescence image used to target the specific OHC for vibrometry measurements. This image was used to target the range of x-coordinates (pixels) in the B-scan image that contained the OHCs. Panel (c) shows the normalized intensity plot of the A-line that follows the vertical dashed line in panel (a), with the horizontal dashed line representing the threshold value used to determine if the displacement measurements would be used or not. Panels (d) and (e) show the vibration amplitude and phase, respectively, of the thresholded A-line along depth. Panels (f) and (g) show the vibration amplitude taken from the BM location and the OHC regions represented by the red and yellow $X^{\prime }$s in panel (a).

As a telling final test of the system capabilities, we noninvasively collected vibrometry data from the cochlea of a Myo15CreXAi6 mutant mouse through the round window membrane using a 70 kHz stimulus frequency at 90 dB sound pressure level. We used TPM to localize the OHCs and then measured the vibration of the basilar membrane (BM) and a single OHC in the same A-line represented by the white dashed line in the cross-sectional (B-scan) OCT image in Fig. 6(a).

The entire A-line, shown as the dotted line in the B-scan in Fig. 6(a), was processed for the vibrometry data to produce the rest of the plots found in Figs. 6(c)–6(g). Figure 5(c) shows the intensity along the A-line that is indicated by the dotted line in Fig. 6(a). This intensity plot has been thresholded to remove low intensity (low SNR) pixels where the displacement data are not trustworthy. Here, any displacement measurement from a pixel with an intensity that was below the average intensity noise plus one standard deviation was removed and ignored in the subsequent plots. Figure 6(b) shows the enface TPM image of the hair cells that was used to localize the correct x-location of the hair cells in Fig. 6(a). The horizontal white dotted line shows the path that the laser was scanned across the tissue when collecting the B-scan image, shown in Fig. 6(a), and the vertical dotted lines indicate the left and rightmost boundaries in the x-direction that would contain the OHCs.

The vibrometry measurements were further thresholded to show only the pertinent displacement information that was collected well above the noise floor of the phase. The phase noise for this dataset was found to be 0.3067±0.1538  nm in the frequency bins adjacent to the 70 kHz stimulus frequency. Thus, any vibration amplitude below the average phase noise plus three standard deviations of the phase noise (0.7681 nm in this case) was set to zero.^18^ Both the BM and OHC regions had a vibrational amplitude well above this threshold.

As was expected, vibrations were largest at the basilar membrane and decreased with A-line depth toward the OHCs and reticular lamina. All regions moved in phase with the only deviation being less than a quarter cycle. For a 70 kHz stimulus, the vibration amplitude of the BM in Fig. 5(f) was found to be 2 nm, whereas the vibration amplitude of the OHC in Fig. 5(g) was 25% lower at 1.5 nm. These plots were selected from the vibration measurements at depth locations corresponding to the red and yellow X’s seen in Fig. 6(a). They show that the vibration measurements at both locations were well above the noise floor—providing visual confirmation that the value of each of these measurements could be trusted.

**Fig. 7:**
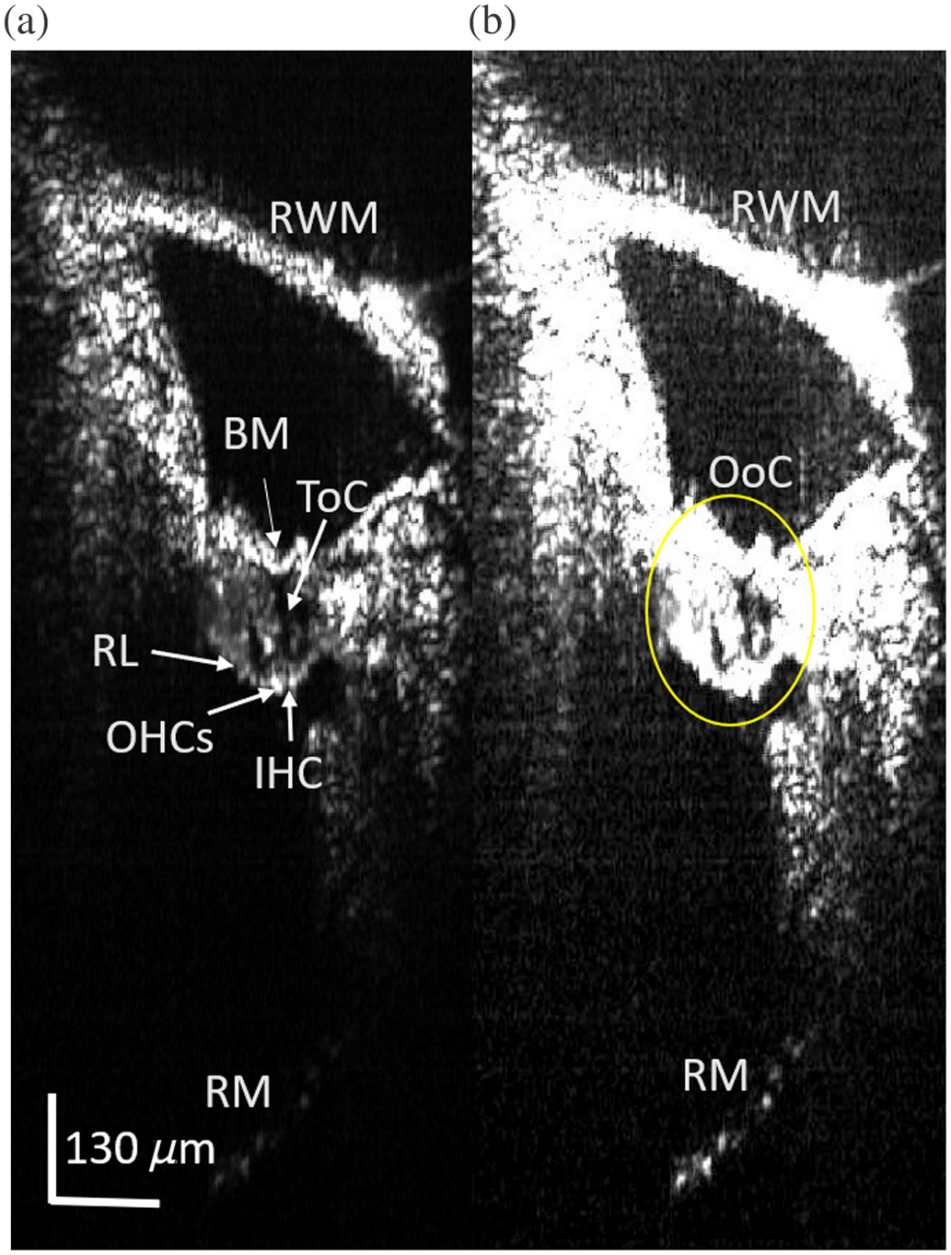
(a) Averaged B-scan image showing the RWM, RM, and the OoC. Within the OoC, we can also see structures such as the reticular lamina (RL), OHCs, ToC, and BM all labeled with an arrow pointing them out. Panel (b) shows the same image with the upper region saturated to better visualize RM at the bottom of the image.

To study the effects of blast or noise trauma, we need to have the ability to image Reissner’s membrane and the OoC to resolve ELH. ELH is characterized by a distension of Reissner’s membrane. In such an experiment, we would expect the user to image Reissner’s membrane periodically by simply switching from a high-power objective to the 4× objective, taking several frames of cross-sectional images, and switching back to the high-power objective for vibrometry measurements or high-resolution imaging of the OoC. In Fig. 7(a), we show an averaged, representative B-scan taken through the round window membrane (RWM) at the end of the *in vivo* vibrometry experiment. We averaged 400 B-scan frames that were taken over 1 s of imaging time to boost the SNR and provide a clearer image of the mouse OoC through the RWM. In this image, we can see the RWM, OoC, and Reissner’s membrane, which, from this viewing angle, is deeper than other areas of the cochlea. We can also see smaller structures that make up the OoC, e.g., the OHC region, the IHC, BM, RL, and the ToC. Reissner’s membrane appears dim in Fig. 7(a) because of the dynamic range of the image in grayscale. In Fig. 7(b), we saturated the bright OoC to make Reissner’s membrane more visible. Nominally, we were focused on the OoC for these images, so if the desired target for imaging was Reissner’s membrane, e.g., to look for ELH, then we would focus deeper and further improve on the image quality.

## 4 Conclusion

Dynamic imaging of the cochlea at cellular resolution is challenging. We need the capability to survey the anatomy at low NA to find the area of interest and then switch to high NA to resolve cellular features such as the hair cells and nerve fibers. Using OCM for the survey mode reduces the photobleaching of the transgenic fluorescent protein labels. The co-registered OCM and TPM images let us take advantage of the molecular contrast afforded by fluorescent labels and compensate for the low contrast in OCM (e.g., Fig. 4). The molecular contrast allows us to measure the vibratory response with OCM at any position within the OoC and have confidence that we are targeting the intended structure (e.g., Fig. 6). With the development of this microscope, we are poised to make detailed investigations of the complex signal transduction process within the mouse cochlea.

## Data Availability

Supporting data for this work are available upon request.
